# Current Advances in the Functional Diversity and Mechanisms Underlying Endophyte–Plant Interactions

**DOI:** 10.3390/microorganisms12040779

**Published:** 2024-04-11

**Authors:** Caihong Zhao, Johnmark Onyino, Xiquan Gao

**Affiliations:** 1 State Key Laboratory of Crop Genetics & Germplasm Enhancement and Utilization, Nanjing Agricultural University, Nanjing 210095, China; 2021101074@stu.njau.edu.cn (C.Z.); onyinojohnmark@gmail.com (J.O.); 2 Collaborative Innovation Center for Modern Crop Production Co-Sponsored by Province and Ministry, Nanjing 210095, China; 3 College of Agriculture, Nanjing Agricultural University, Nanjing 210095, China

**Keywords:** endophytes, phenotype, host–endophyte interactions, disease triangle

## Abstract

Plant phenotype is a complex entity largely controlled by the genotype and various environmental factors. Importantly, co-evolution has allowed plants to coexist with the biotic factors in their surroundings. Recently, plant endophytes as an external plant phenotype, forming part of the complex plethora of the plant microbial assemblage, have gained immense attention from plant scientists. Functionally, endophytes impact the plant in many ways, including increasing nutrient availability, enhancing the ability of plants to cope with both abiotic and biotic stress, and enhancing the accumulation of important plant secondary metabolites. The current state of research has been devoted to evaluating the phenotypic impacts of endophytes on host plants, including their direct influence on plant metabolite accumulation and stress response. However, there is a knowledge gap in how genetic factors influence the interaction of endophytes with host plants, pathogens, and other plant microbial communities, eventually controlling the extended microbial plant phenotype. This review will summarize how host genetic factors can impact the abundance and functional diversity of the endophytic microbial community, how endophytes influence host gene expression, and the host–endophyte–pathogen disease triangle. This information will provide novel insights into how breeders could specifically target the plant–endophyte extended phenotype for crop improvement.

## 1. Introduction

Plants supply an ecological niche that harbors diverse species including those that form the plant microbiome, regarded as an extension of the plant genome. This microbial community also extends the plant phenotype [1]. The significance and mechanism of the bidirectional relationship between plants and endophytes have left the scientific community with a puzzle to crack, with intentions to explore the plant’s extended phenotype [2,3,4,5]. Endophytes play indispensable roles during plant growth and development by jacking up the host plant defense against phytopathogens, which is accomplished by augmenting phytohormone production, such as that of salicylic acid (SA), jasmonic acid (JA), and ethylene (ET), increasing leaf toughness through cellulose deposition, protecting plants from herbivorous insects, niche competition, and producing hydrolytic enzymes [6,7,8,9,10]. Furthermore, they also patch up the host plant’s ability to adapt to abiotic stresses and increase nutrient availability [6,11]. In return, plants provide the endophytes a livable environment and essentials like water and nutrients for their growth [12].

Endophytic communities are intricate formations comprising bacteria or fungi from diverse taxa [13,14,15,16]. The host genotypes, the host plants’ locale, and environmental selections constitute some of the elements that might influence the assembled endophyte structure [17,18,19,20]. Before being recruited by the host, endophytes may exist as free-living microorganisms in the external environment, such as in the rhizosphere, and then they are recruited, activated, and assimilated to become part of the microbial flora within the host plant [21]. On the other hand, the host may selectively inherit microorganisms that can confer excellent traits to the offspring; this forms a core group of endophytes [22]. Eventually, core groups of endophytes associated with traits such as disease resistance [19] might be passed as a “legacy” directly from mother plants to their offspring.

Genetic signatures from both endophytes and host plants have been implicated in controlling the existence of this interrelationship. Endophytes induce the expression of specific genes in plants, for instance those involved in the production of specific metabolites or those that help the plant cope with environmental stress [23,24]. On the other hand, plants modulate the colonization and subsequent survival of endophytes in a complex but unique manner from how they respond during phytopathogen invasion [25,26,27]. Furthermore, studies based on phenotypic differences between plants inhabiting a particular taxon of endophytes and those that do not show that genetic cues could control the plant–endophyte relationship [28]. 

There are multiple previously published excellent review works focusing on the interaction between endophytes and host plants [29,30,31,32,33]. The potential of endophytes in crop improvement has also been fully discussed [34,35,36]. However, there exists a knowledge gap in how genomic fingerprints of endophytes influence key host plant regulatory pathways and the associated host microbial community phenotype. It is also imperative to explore the genetic mechanisms of how plants influence and assemble associated endophyte communities. 

This review attempts to summarize the most recent advances in the tripartite endophyte–plant–pathogen interactions, highlighting the genetic mechanisms of endophyte–host interaction from colonization to symbiosis. This will provide insights into how plant scientists and breeders could focus on plant genetic regions that modulate endophytes as an extension of the host plant phenotype. Thus far, breeding for plants to exclusively express favorable extended microbial phenotypes will not only increase yield but also help plants withstand the negative effects of various abiotic and biotic stresses, eventually contributing to sustainable agriculture.

## 2. Systemic and Non-Systemic Endophytes

### 2.1. Definition of Systemic and Non-Systemic Endophytes

In 2011, Botella et al. classified endophytes into two categories, systemic and non-systemic endophytes, based on factors such as phylogenetic relationships, metabolic potential, physiological characteristics, and modes of transmission [37]. Systemic endophytes are those that have co-evolved with the host plant during the evolution process, share the host’s metabolic and genetic composition [38], and are resistant to the host’s immune system [39]. The systemic endophytes usually do not harm the host at any stage of their life cycle, or cause disease, and can even be inherited from the host plant to subsequent generations (through seeds or vegetative propagules), also known as true endophytes [3,40,41]. Corresponding to this are the non-systemic endophytes that live asymptomatically for at least part of their life cycle in plant tissues. However, they are potential pathogens, in case host living conditions turn unfavorable [37].

### 2.2. Comparative Genomics of Systemic Endophytes and Non-Systemic Endophytes

To investigate the differences between systemic endophytes and non-systemic endophytes, comparative genomics and metabolic network studies (involving 36 non-systemic endophytes and 28 systemic endophytes) were conducted, which revealed that non-systemic endophytes and systemic endophytes differ in terms of metabolic capabilities and cellular processes [28]. Previous research work has indicated that while non-systemic endophytes have more genes related to breakdown and host invasion, systemic endophytes have more biosynthetic genes [28]. In addition, non-systemic endophytes and systemic endophytes are marked with unique expression patterns in their genomes. The former has a putative novel secretion system with circadian rhythm regulators, and the latter encodes nitrogenase and ribulose diphosphate carboxylase/oxygenase (RuBisCO) genes. Remarkably, genes linked to the metabolization of plant stress-related compounds were also prevalent in systemic endophytes [14]. Zaluga et al. identified that endophytic *Clavibacter* LMG 26808 lacks the most important virulence factors of the pathogenic *Clavibacter* strain. Also, the number of genes encoding extracellular proteins and digestive enzymes were fewer in the symbiotic endophytes as compared with the number of genes encoding transport proteins and transcriptional regulators [42]. 

Of important note is that separate comparative analyses of genomes of several common systemic endophytes have revealed that beneficial systemic endophytes are not restricted to a putatively distinct pattern and have a wide variety of lifestyles [43]. The aforementioned evidence shows that systemic endophytes have continuously evolved their lifestyle to adapt to different environmental conditions within the plant in a manner distinct from that of non-systemic endophytes.

## 3. Impact of Endophytes on Host Plants

### 3.1. Promoting Plant Growth

#### 3.1.1. Enhancing Nutrient Availability

As is widely acknowledged, endophytes have essential functions in enhancing plant nutrition. Endophytes increase the availability of plant nutrients by directly synthesizing and releasing them to plants for use or enhancing host plant synthesizing pathways. Increased nutrient availability could be used to feed the endophytes. In return, plant biomass accumulation will be increased and plant growth will be promoted.

Legume-specific rhizobia possesses the unique ability of symbiotic nitrogen fixation, while nitrogen-fixing endophytic bacteria have also been identified in non-nodulating plants, such as sugarcane [44,45]. Within a plant’s physiological system, nitrogen-fixing endophytes help convert atmospheric dinitrogen gas (N_2_) into nitrogen compounds that are accessible to plants, such as ammonium [46,47]. In a study using finger millet, two fungal endophytes, *Aspergillus terreus*, and *Lecanicillium* sp., and two bacterial endophytic isolates *Pseudomonas bijieensis* and *Priestia megaterium* were identified, which showed strong promotive effects on NPK contents in grains [48], further supporting the role of endophyte in nutrient enhancement in the host.

Additionally, nitrogen cycling-associated bacteria have the potential to enhance plant nitrogen use efficiency, thereby expediting the process of nitrogen conversion [11,44]. Endophytes can also produce siderophores, which are organic compounds secreted by microorganisms and plants under iron-limited conditions. These siderophores can bind to iron in the environment, which is absorbed by host plants [14,49]. Furthermore, some endophytes can convert phosphate from the soil into ortho-phosphate by releasing organic acids into the soil, which are readily absorbed by plants [50].

**Figure 1:**
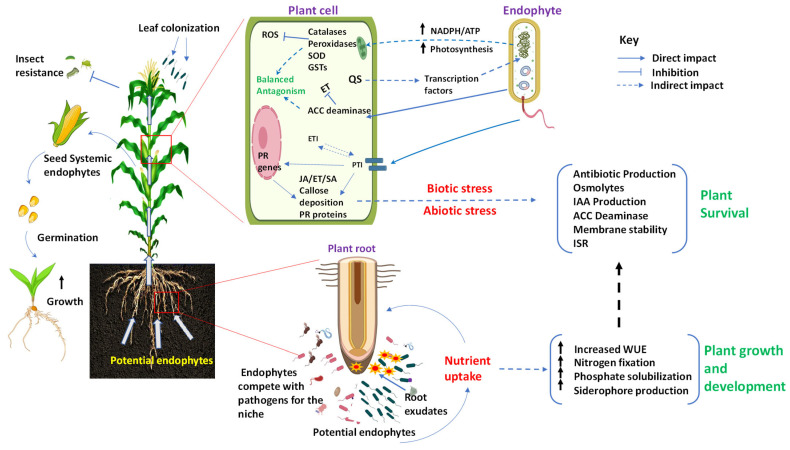
Proposed model for endophyte colonization of plant host through balanced antagonism. Endophytes living close to the host plant organs can be recruited by the host plant horizontally from roots and the air or vertically through propagation materials such as seeds. Through balanced antagonism, endophytes and host plant maintain a balance for coexistence. The endophytes increase nutrient availability and uptake, as well as photosynthesis, which promotes plant growth and development. Moreover, the ability of the host plant to resist plant pathogens and insects and survive various abiotic/biotic stresses is enhanced through the priming of the host defense by endophytes and the induction of pathogen-related genes and/or phytohormones. (ET, ethylene; SA, salicylic acid; JA, jasmonic acid; ACC, aminocyclopropane-1-carboxylic acid; QS, quorum sensing; WUE, water use efficiency; ISR, induced systemic resistance; IAA, indole acetic acid; GSTs, glutathione-S-transferases; SOD, superoxide dismutase; PTI, PAMP-triggered immunity; ETI, effector-triggered immunity; ROS, reactive oxygen species; PR, pathogenicity-related).

On the other hand, endophytes can improve plant photosynthetic capacity by enhancing the levels of chlorophyll pigments, and chloroplast metabolism [51], as well as NADPH/ATP supply (Figure 1), which are needed for carbon assimilation [52]. Inoculation of tested Chenopodium sprouts with *Streptomyces* (strain JSA11) resulted in significant increases in photosynthetic activity and respiration rate due to significant increases in chlorophyll a and b [52]. In another study, sugar beet plants inoculated with endophytes were found to accumulate higher total carbohydrates than the control plants [51]. Endophytic inoculation has been reported to increase amino acid availability, such as that of asparagine, threonine, serine, glutamine, alanine, etc., in mustard plants [53]. It can be hypothesized that endophytes increase plant nutrient supply to avoid overwhelming the host’s metabolic resources. Despite research progress on metabolic enhancement by endophytes toward host plant nutrient enrichment, there are still research gaps in targeting the influence of endophytes on host gene expression and the pathways involved (Figure 1). Digging more into this area could provide eco-friendly approaches to increasing crop biomass and yield.

#### 3.1.2. Promoting the Biosynthesis of Phytohormones

Plant hormones play important roles in many aspects involving plant growth, development, and stress responses. Endophytes promote plants’ growth via the production of phytohormones within the host plant [9]. In particular, some endophytes, for instance, synthesize indole-3-acetic acid (IAA), favor plant growth, and increase total root surfaces to enhance the absorption of nutrients from the rhizosphere soil [54,55]. Certain seed-borne endophytes strains, such as *Bacillus amyloliquefaciens* RWL-1 in rice [56], and leaf-borne endophytes such as *Psuedomonas resinovorans* and *Paenibacillus polymaxain* produce gibberellin (GAs) and cytokinins (CTKs) [57]. In another study, it was determined that *Piriformospora indica* lessened the juvenile phase in black pepper by triggering the independent GA biosynthesis, photoperiodic, and age pathways, which subsequently encouraged growth and preponed floral induction [23]. *B. amyloliquefaciens* FZB42 enhanced systemic salt tolerance by upregulating the expression of two lipoxygenases (*AT1G17420 and AT1G72520*) and one allene oxide cyclase (*AT3G25780*), which are important genes for the biosynthesis of JA, when treated under salt stress conditions as opposed to when not inoculated. Furthermore, four genes involved in JA synthesis were also upregulated by FZB42 under non-stress conditions, whereas jacalin lectin family protein (*AT1G52100*) and jasmonic acid carboxyl methyltransferase (*JMT*), a key enzyme for jasmonate-regulated plant responses, were downregulated [58]. These findings provide clear evidence that, for endophytes to successively coexist with the host plants, targeting essential pathways involved in the synthesis of these signaling molecules and the signal activation of various gene clusters involved in the synthesis of particular metabolites are important means of communication. Remarkably, the above studies have demonstrated that endophytes augmented host phytohormone supply (Figure 1), placing a premium on the coexistence and evolutionary interaction between endophytes and their respective host plants.

### 3.2. Enhancing Stress Tolerance and Disease Resistance

#### 3.2.1. Biotic Stresses

Plants may encounter diverse biotic stresses throughout their growth, such as herbivore grazing and pathogen infection [10]. Endophytes prime various host defense pathways, through the release of antimicrobial compounds, production of siderophores, and induction of plant systemic acquired resistance response [59,60,61,62]. Endophytic fungi derived from *Eucalyptus exserta* possess the ability to secrete secondary metabolites with antimicrobial properties against *Ralstonia solanacearum*, thereby serving as potential alternatives to conventional antimicrobials [8]. Some bacterial endophytes can synthesize insecticidal alkaloids or phenols such as azadirachtin, which aid the host in combating insect gnawing [63]. Intriguingly, in a different study, the root symbiotic endophytic fungus *Phomopsis liquidambaris* showed a strong inhibitory effect on Rice spikelet rot disease (RSRD) caused by mycotoxins produced by the pathogen *Fusarium proliferate*. This role is attributed to the alternation of spikelet microbiome composition, among which *Pseudomonas* and *Proteobacteria* having resistance activity were significantly enriched, likely through enhancing the production of numerous secondary metabolites, especially hordenine and l-aspartic acid in rice spikelets [64]. 

The colonization process of host plants by certain endophytes can elicit plant defense responses that resemble those triggered by beneficial microbes, e.g., induced systemic resistance (ISR) [8,65,66]. In connection to this, inoculation of *Arabidopsis thaliana* with the beneficial bacterium *B. cereus* AR156 prompted induced systemic resistance (ISR), which is NPR1-dependent and mediated through the JA/ET signaling pathways via increasing expression levels of defense-related genes (*PR1*, *PR2*, and *PR5*) and mitogen-activated kinase (MAPK) cascade marker gene *MPK6* [24]. 

When plants are subjected to stress, ET signal transduction pathways are activated as a coping mechanism. However, the persistent accumulation of ethylene hampers normal plant growth and development, even resulting in plant mortality. Endophytes possess the ability to produce the 1-aminocyclopropane-1-carboxylic acid (ACC) deaminase enzyme, which impedes the ET biosynthesis pathway in plants, thereby mitigating its excessive buildup, hence balancing plant growth and stress response [67]. Interestingly, when genes involved in ET biosynthesis are mutated in plants, endophytes also possess the capability to secrete ET as a compensatory mechanism against the mutation’s impact on the plant phenotype [68]. It is elucidated that this phenomenon can be ascribed to the accelerated evolution of short-living endosymbionts within longer-living hosts, thereby facilitating the development of more optimal adaptations to diverse stresses and aiding their hosts in withstanding external pressures [14].

Endophytes prime the host defense in a complex manner that could typically hinder their coexistence with host plants. The endophytic metabolic capabilities and influence on host immune gene expression/pathways are the unique factors of divergence from the pathogenic members for instance, selective downregulation of the *SHR5* gene during endophytic colonization in sugarcane [69]. Further research is warranted to shed more light on the specificity with which plants perceive colonization by endophytes and the genetic mechanism by which ISR is triggered against pathogen invasion.

#### 3.2.2. Abiotic Stress

Plants are often exposed to extreme environmental conditions, including salinity, flood, drought, and temperature (low/high) [6]. The presence of endophytes can ameliorate the impacts of multiple stresses on plants through diverse mechanisms, including phytohormone production, secondary metabolite production, the induction of host genes, and ROS scavenging activity [65,66,70,71,72,73,74,75,76,77,78]. Endophytic fungus *P. indica* increased the expression of *P5CS* and *TAS14* increased drought stress tolerance in plants [74]. Likewise, in sunflower (*Helianthus annuus* L.) and soybean (*Glycine max* L.), the endophytic fungus *Rhizopus oryzae* reduces heat stress and encourages plant growth [72]. *B. amyloliquefaciens* FZB42 relieved sensitivity to salt stress in Arabidopsis [60]. It can be summarized that, to engage the tolerance mechanism and help withstand the abiotic stress conditions, endophytes produce metabolites and receive signals that either activate genes that respond to stress or genes that initiate cross-talk with target stress-tolerant genes.

## 4. Regulation of Endophytes by Host Plant

### 4.1. The Recruitment of Endophytes Regulated by Plant

#### 4.1.1. Rhizosphere Metabolites from Host Plant

Before being recruited by the host, endophytes may exist as free-living microorganisms in the external environment, such as in the rhizosphere (Figure 1). During recruitment, they are activated and assimilated to become an integral part of the microbial flora within the host plant [21]. Plant roots are considered to be the key initiators of plant-microbe interactions in the soil, attracting bacteria to the interior of the roots through rhizosphere exudates [18,21,79,80,81]. The content of rhizosphere exudates mainly includes carbohydrates and amino acids, which are key signaling molecules between host plants and endophytes. Root exudates can support the assembly and maintenance of specialized microbiota tailored to the needs of the host [82,83,84,85]. For example, oxalate is involved in the host plant recruitment of *Burkholderia phytofirmans* [86]. Furthermore, different host plants exhibit unique secretion patterns, which could be the sole reason for host plant species specificity and the associated microbiome [83]. In addition, plant rhizosphere metabolites fluctuate based on the plant’s stage of growth and development. This is in turn associated with precedented changes in the rhizosphere microbial community due to microbial substrate absorption preferences. Since gene expression patterns of host plants are involved in these changes [84], it is possible to make predictions based on plant genome information.

It is also evident that endophytes possess specific chemotactic capabilities, which comprise the major medium utilized by plants to signal the swimming of potential endophytes and subsequent colonization within host plant tissues. It is interesting to know how particular exudates selectively target particular endophytes. In this context, it has been demonstrated that the same chemotactic attractant can exhibit different patterns among not only genera but also species and even strains of the same species. This shows that the dominance of plant root exudates extends the gene expression profile of targeted endophytes, as demonstrated by fumaric acid, which increased cheA and biocontrol-related gene transcript levels, and in turn, strongly boosted *B. amyloliquefaciens* subsp. *plantarum* YP1’s ability to chemotaxize and thrive [87].

Understanding the genetic patterns that manipulate endophytic root recruitment will provide insights into how targeted endophyte recruitment can be utilized in crop improvement, for example by linking endophytic GWAS (genome-wide association study) analysis with particular plant pathways synthesizing these exudates. It is also noteworthy that the most prevalent form of endophyte recruitment, which is currently evidenced by the obvious abundance of endophytes in soil, is through root exudates.

#### 4.1.2. The Vertical Transmission of Endophytes from Plant

Endophytes can also be transferred vertically from parents to offspring through propagation materials such as seeds (Figure 1) or vegetative propagules [22,88,89]. In most cases, seeds are a good source of host genotype-specific endophytes [89,90]. In a study utilizing 500 genotypes of the temperate forage grass *Lolium perenne*, the vertical transmission of endophyte strain AR37 of *Epichloë festucae var. lolii* was systemically investigated. Interestingly, it was found that both host genotypes and environmental conditions significantly affect AR37 transmission in seeds [90]. Another study utilizing corn kernels identified differences in the abundance and type of endophytes based on genotype specificity. Surprisingly, the endophytic bacterial community in the parental seeds and their genetically related offspring seeds had a similar composition, especially the genera-dominant composition. This phenomenon may be caused by either heredity or the parent and offspring having the same growth environment [91]. A small core group of microorganisms is usually maintained at a high relative abundance regardless of the growth stage of the host plant or environmental factors [92,93,94,95]. This conserved core microbiota is linked to the evolution, domestication, and migration of maize [96]. The seed-borne endophyte in rice can be cross-generationally transmitted to shape the disease resistance of rice [19]. In addition, seed endophytes are also beneficial for host plants to grow and develop in harsh environmental conditions [22]. Therefore, through the long co-evolution process of plants and their microbiomes, microbiome self-regulation has been developed based on selective feedback from the host plant.

Other studies utilizing Arabidopsis [82,97], foxtail millet [98], sugarcane [99], tangerine [100], and coconut tree [84] identified overlapping microorganisms in the different microbiomes of these different plants. This indicated that plant genes controlling plant-associated microbial diversity might be similar between different plant species. Additionally, the recruitment of endophytes is influenced by various intrinsic factors within the plant itself. Currently, the prevailing consensus suggests that soil serves as the primary source of endophytes in plants. However, further investigation into the vertical transmission of endophytes is warranted, as these cross-generational transfers may be subject to regulation by plant genes. Plant development and breeding for resistance may benefit from cross-generational endophytes, which could be important microbial resources.

### 4.2. The Colonization and Growth of Endophytes Regulated by the Plant

#### 4.2.1. Regulation of Plant Immune Response during Endophyte Colonization

The attachment of microorganisms in the soil to the interior of plants is considered an initial and crucial step in facilitating plant-microbe interactions. The location of endophyte colonization in plants was once controversial; nevertheless, with the advancement of technology, it is now possible to track and visualize the colonization patterns and orientations of endophytes. For instance, laser confocal microscopy can be used to identify areas of high colonization, and fluorescence in situ hybridization (FISH) can be used to study colonization pathways and track key individual endophyte taxa; therefore, multiprobe arrays can define an endophyte community structure [101,102,103].

Plants have evolved two innate immune systems, e.g., pattern-triggered immunity (PTI) and effector-triggered immunity (ETI), to combat the invasion of various pathogens. PTI and ETI exhibit significant distinctness in terms of recognition mechanisms and early signal transduction; however, they also demonstrate a synergistic interaction pattern within their immune responses [104,105,106,107]. Although plants have evolved complex and relatively comprehensive immune systems, some microorganisms can still bypass plant defense responses. Nonetheless, the plant immune system plays an active role in recognizing the colonization of symbiotic microorganisms [25,97]. Because ligand-receptor interactions determine the microbial attachment steps, plants can respond differently to invasion by endophytes or pathogens, whereby microbe-associated molecular patterns from endophytes do not trigger a strong immune response in plants [108]. For instance, the grapevine flagellin receptor VvFLS2 (Flagellin-Sensitive 2) exhibited differential recognition toward endophytic bacteria that promote plant growth and pathogenic bacteria that induce plant diseases [26].

Mild defense responses by plants induced during endophyte colonization activate plant-induced systemic resistance. Plant defense genes are expressed with a modest oxidative burst during endophyte colonization, and numerous pathways, including protein methylation and phytohormone responses, are transcriptionally activated [26]. Contrarily, the induction of defense proteins or cellular detoxification is triggered when pathogenic microbes possessing a lot of plant cell wall-degrading enzymes (PCWDEs) invade host plants [108,109,110,111,112]. Martin et al. speculated that symbiotic endophytes have evolved capabilities that do not fundamentally damage plant tissues during colonization; hence, a strong immune response is not triggered by the plant host [113]. Fascinatingly, a recent study on the population dynamics of bacterial endophytes (*Achromobacter xylosoxidans* and *Pandoraea* spp.) discovered that the long-term stability of the endophytic phyllosphere microbiota is maintained by the population density of non-pathogenic members due to an equilibrium between bacterial reproduction and mortality. This finding may suggest that plants can actively regulate the population density of endophytes [27].

The modulation of host immune-related pathways is quite complex, and for a balanced antagonism to be achieved, downregulation or upregulation of gene expression of particular host defense signaling pathways may be induced by endophytes, particularly by, for example, turning off Gibberellins (GAs) response gene expression or inducing late SA/JA/ET signaling and genes targeted by miRNAs to secure successful proliferation and colonization by endophytes [111]. Noteworthy is the fact that endophytes can metabolize potential MAMPS that could otherwise pose as potential pathogens. The perception of flagellin via FLS 2 from endophytes is also different from that of phytopathogens, and chitin-specific receptors (PR-3) of plants that recognize chitin oligomers, for example, are unable to recognize endophytes when chitin deacetylases chitosan oligomers [114]. Future research should target the endophyte-host immune interaction and dig more into the mechanisms of endophytes before, during, and after their colonization by analyzing the gene expression patterns of endophytes during these stages to identify potential genetic manipulation and signaling by host plants.

#### 4.2.2. The Nutrient Assimilation and Hormone Metabolism of Plant

The host plant’s nutrient uptake can also affect endophyte colonization [115]. In Arabidopsis, the phosphate status of the host affects the colonization ability of the root endophytic fungus *Colletotrichum tofieldiae* [116]. Host plants physiologically and biochemically respond distinctly depending on the type of endophytes [30]. Specifically, plant hormones influence endophyte development and metabolism after colonization. The abnormal distribution of root microbes is attributed to the functional impairment of three phytohormones and their biosynthesis genes, including SA (involving *CPR1*, *SNC1*, *PDA4*, *SID2*, and *NPR1* et al.), JA (involving *JAR1*), and ET (involving *EIN2*). Notably, this alteration is not achieved by modulating the abundance of a small dominant strain in each differentially abundant family [117]. The elevated concentration of auxin may impede the proliferation of plant-associated microorganisms, including bacteria belonging to the *Pseudomonas* genus, which are some of the most common endophytes in plants [118]. The plant hormone abscisic acid (ABA) is postulated to activate specific receptors in the endophytic fungus *Aspergillus nidulans*, thereby inducing modifications in gene transcription, protein synthesis, and degradation within the central cellular machinery, particularly affecting processes such as translation, transcription, and glycolysis. Ultimately, ABA modulates the biosynthesis of secondary metabolites and fungal toxins produced by *A. nidulans* [119].

#### 4.2.3. Host Tissue Specificity of Endophytes

Endophyte colonization also has tissue specificity [82,120]. A study investigating the composition of endophytic microbial communities in both roots and leaves of Arabidopsis revealed that specific genotypes of Arabidopsis exhibited unique leaf and root microbiota, indicating that tissue-specific genotypes exerted varying effects on endophytic bacteria in both roots and leaves, thereby significantly influencing community composition [82]. Notably, although the host’s genotype determines its microbiome, the host plant’s genetic control of endophytes in aboveground tissues is stronger than that in belowground tissues [82]. When exploring plant genotype–endophytic microbial relationships, it is necessary to consider the fact that the soil environment will increase the complexity of microbiome assembly. Similar research works have also found a correlation between endophytic microbial community composition and plant host phylogeny in maize [121], Arabidopsis [122], and some other terrestrial plants [84]. Both tissue type and plant group have an impact on the prospective endophyte community or population. Host tissue specificity could be interconnected to the vertical transmission of endophytes due to systemic growth in specific reproductive parts of host plants.

### 4.3. The Community Structure of Endophytes Regulated by The Plant

#### 4.3.1. Physical Barrier Formation Pathways in Plants

The plant cuticle is waxy and largely composed of polyester cutin. It plays a role in protecting the plant from pathogens and water loss. On the other hand, it serves as a key contact factor for plant microbiome interaction including endophytes. A study on Arabidopsis leaves revealed that the diversity of leaf-associated microbiota was influenced by the composition of cuticular wax components and their permeability [123]. Interestingly, a different study found that the host genes *LACS2* (*Long chain Acyl-Coenzyme A Synthetase 2*) and *PEC1* (*Pectin 1*), closely associated with cuticle permeability, exerted the most pronounced influence on phyllosphere microbiota [124]. The synthesis of waxes in the plant leaf epidermis involving *CER1*, *CER6*, *CER9*, etc. (*CER*, *ecerfiferum*), influences the composition of the phyllosphere microbial community [125]. These data suggest that the increased permeability of the cuticular wax is advantageous for the proliferation of endogenous bacteria, potentially due to a positive association between cutin permeability and nutrient availability [126]. Recent research has also discovered that the regulatory network controlling the diffusion barrier of endodermal roots also impacts the composition of root-associated endophytic fungal communities, indicating that plant physical barrier formation pathways play a role in plant-microbe interactions [126]. The study also demonstrated that root-associated endophytes can exert an influence on the functionality of the root diffusion barrier in a reciprocal manner. Thus, the study proposed that the coordination between the root diffusion barrier and the microbiome facilitates plants’ adaptation to fluctuations in mineral nutrient availability in natural environments, thereby enhancing their survival capabilities in extreme habitats. These studies suggested that the interplay between plant barriers has a crucial role in nutrient uptake and the colonization of endophytes. Consequently, the coordination of plant barrier functionality with the microbiome could potentially enhance plant stress resistance, yield, and overall quality, according to the needs.

#### 4.3.2. Innate Immune Pathways in Plants

The intricate function of plant innate immune pathways in controlling the endophyte community may be influenced by a range of pleiotropic factors. It is suggested that the signaling pathway triggered via the single recognition of microbe-associated molecular patterns (MAMPs) through their perception by pattern recognition receptors (PRRs) might not significantly impact the composition of the plant endophytic microbiome, yet represents only one aspect of the multi-level interactions between plant innate immune systems and endophytes [127]. It was found that in Arabidopsis, pattern-triggered immunity (PTI) and the MIN7 vesicle-trafficking pathway are involved in shaping the endophytic microbial community in the phyllosphere, and *constitutively activated cell death 1* (*CAD1*) could be a potential convergent component of PTI and the MIN7 vesicle-trafficking pathway [128]. Therefore, plants can modulate the composition and abundance of endophytic microbiota through the genetic pathways involved in immunity to regulate the dynamic equilibrium of endophytes, thereby establishing a climate-resilient microbial community and fostering an optimal internal environment for plant health. 

There is also evidence to suggest that there are functional networks responsible for plant stress responses, immune system functionality, and the assembly/maintenance of the microbiome intersect in plants; specifically, host plants can regulate their associated microorganisms by modulating innate immune systems [79]. Notably, this work identified a pivotal transcription factor, PHR1, which integrates the output of the plant immune system and the phosphate starvation response (PSR), contributing to the assembly of the normal root endophytes. Under drought stress conditions, plants might suppress their immune response to alter the composition of root endophytic bacterial communities [129,130]. These findings suggest that plants are capable of controlling microbial assembly, in conjunction with other physiological pathways within plants, likely through constituting intricate and cohesive gene-regulatory networks. Therefore, plants can modulate the composition and abundance of endophytic microbiota through the genetic pathways involved in immunity to regulate the dynamic equilibrium of endophytes, or on the other hand, alter endophyte composition to further modulate host innate immunity, thereby establishing a climate-resilient microbial community and fostering an optimal internal environment for plant health. Further investigation into the role of plant immunity within the intricate regulatory networks involved in plant–microbial interactions will not only advance our comprehension of these systems, but also facilitate the design of synthetic microbial communities (SynComs) that can benefit field production.

#### 4.3.3. Metabolic Pathways Involved in Plant Secondary Metabolite Biosynthesis

Plants could modulate metabolite synthesis to shape and select particular endophytes [25]. The research on chemical defense in Arabidopsis flower tissues discovered that two co-expressed clusters of terpene synthases and cytochrome P450-encoding genes (*TPS11* and *CYP706A3*) play a role in producing terpenoid metabolites that impact the assembly of specific bacterial communities colonizing Arabidopsis flower tissues [131]. Plants produce flavonoids as a physiological reaction to stress, but they also draw microbes, demonstrating flavonoid chemotaxis in the relationship between microbes and plants [132,133]. Arabidopsis roots improve the structure of rhizosphere microbial communities by secreting coumarin, which represents a specialized class of secondary metabolites with potent antibacterial activity against phytopathogen [134]. The study of root tissues also revealed that the plant synthesizes a range of specialized triterpenoid metabolites and their derivatives through distinct pathways, which play a crucial role in modulating the growth activity of endophytic bacteria and significantly influence the maintenance of microbial communities [135,136]. Moreover, benzoxazinoid compounds exhibiting broad-spectrum resistance against plant diseases and insect pests exert distinct effects on the structure of host-associated microbiota in maize, particularly significantly impacting the abundance of root bacteria [136].

Intriguingly, the dynamic secretion of JA and sugars (specifically, sucrose and trehalose play pivotal roles in early and late stages of development, respectively) by maize root tissues is associated with the shaping of microbial communities at different developmental stages of maize [137]. This study highlights the pivotal role of rhizosphere metabolite sugars in modulating the plant microbiome. Plant-derived carbohydrates generated through photosynthesis, exert diverse effects on rhizosphere microbial communities throughout maize development, with particular significance during early stages as they potentially attract microorganisms from the surrounding soil as a nutrient source. In addition, sucrose has been identified as a signaling molecule capable of inducing root colonization by *Bacillus subtilis* and other potential bacterial species. However, plants can also exert an influence on endophyte chemotactic ability through the production of compounds that disrupt bacterial quorum sensing (QS), which has also been observed in animals [138]. These findings also imply that genetic variation in plant hosts can influence microbial communities in different environments through consistent metabolic pathways associated with disease resistance. This provides a basis for cultivating crops favoring microbes that can be beneficial to host resistance to pathogens, thereby enhancing the growth potential of crops.

#### 4.3.4. Metabolic Pathway of Hormones

Plants employ plant defense hormones, ET, JA, and SA, as the conserved defense mechanism against all microbial colonization attempts, establishing a dichotomy between two distinct microbial lifestyles [20,56]. The immune signal SA, which serves as a carbon source for the partial growth of endophytic bacteria, possesses the capacity to regulate the taxonomic composition of endophytic bacteria at the phylum level, while also exerting direct or indirect effects on the physiology of individual endophytes within specific confined barriers [117]. In rice, metagenomics was employed to identify rice genomic regions governing the abundance of core microorganisms in the microbiome network structure. These specific rice genomic regions are associated with stress responses and carbohydrate metabolism processes, including SA metabolism [139]. Furthermore, it has been reported that the ET signaling pathway involving *EIN*2 (*Ethylene-Insensitive 2*) plays a crucial role in modulating the composition of endophyte communities within the host as well. The loss of EIN2 results in an increased abundance of *Variovorax* cells within the plant, while this mutation has no effect on the abundance of other bacterial species [124]. Interestingly, the metabolism of the plant hormone is also reported to be involved in Arbuscular mycorrhiza fungus (AMF) symbiosis with plant roots. For instance, AMF mycelial development was directly impacted by the upregulation of genes involved in GA (Gibberellin) production (*GA20ox1* and *GA20ox2*) and metabolism (*GA20ox1*) in root tissue cells [140].

### 4.4. The Potential to Confer Novel Functions upon Endophytes

Interestingly, plants can confer endophytes with metabolic abilities to enhance the endophytes’ adaption to the internal environment within the host plant [141]. Horizontal gene transfer (HGT) is an infrequent phenomenon that serves as an alternative mechanism for genetic material exchange events between distantly related species. However, previous studies have found that HGT occurs more frequently in prokaryotes than in eukaryotes [142,143]. Similarly, there is a horizontal transfer of genomic DNA during the interaction between endophytes and plants. During the process of evolution, genetic recombination between endophytes and their hosts may have occurred, resulting in host genes being integrated into endophytes [142].

Due to the low probability of HGT events, there are also fewer reports of HGT events occurring between endophytes and host plants. Pteris vittate (*Pteris vittate* L.)-associated endophytes have been identified to have arsenite transporter *arsB*/*ACR3* genes similar to their host [144]. Other studies in AMF identified 18 genes obtained from plants or bacteria that may play a role in the symbiotic adaptation of this arbuscular mycorrhizal fungus [145]. In the study of genetic breeding for wheat scab resistance, Wang and colleagues discovered that the genome of the temperate grass endophytic fungus *Epichloë* shares similarities with the main scab resistance gene *Fhb7* (*Fusarium head blight 7*) that was previously reported [146]. These findings suggest the potential presence of horizontal gene transfer (HGT) in plants and endophytes, although this hypothesis remains speculative. Given the capacity of certain endophytes to synthesize plant secondary metabolites, it is plausible that plants may have transferred relevant plant genes to their symbiotic endophytes [147,148]. This also implies that horizontal gene transfer events between host plants and endophytes may confer novel functionalities on the latter, thereby facilitating their adaptation to specific ecological niches or augmenting their functions, which bears significant implications in the realms of ecology and biotechnology.

### 4.5. The Interconversion of Non-Systemic Endophytes across Different Life Cycles

The “balance-antagonism” (Figure 1) paradigm governs non-systemic endophytes and plant hosts; in other words, a symbiotic connection occurs when the interests of the two parties are aligned and support each other. However, when the interests of the endophyte cannot be guaranteed, the symbiotic relationship between the two could change [16]. In the cases where the host plant fails to establish a symbiotic relationship with non-systemic endophytes, due to many factors, including the communication of biological signals with the host and external environmental conditions, the developmental stage of the host plant, the external environment, and the host’s defense response [30], these endophytes can be transmitted from symbiosis to parasitism [28,149]. Furthermore, the relationship between host plant immune stability and microbial biomass may influence endophyte lifestyle. In a study utilizing Arabidopsis PTI mutants, it was found that some bacteria with low biomass in Arabidopsis exist as symbionts in wild-type Arabidopsis, but in the PTI mutant they form high abundance and cause damage to the host [128]. Studies on endophytic fungi in grass plants can shed additional light on the harmful and non-pathogenic lifestyle switches made by bacterial endophytes [16].

On the other hand, under the influence of the host, certain pathogenic microbes can transform into non-pathogenic endogenous endophytes. Pathogenic fungi have the potential to transform into non-pathogenic endophytic fungi, which may be influenced by various factors including the host’s genotype and local abiotic stressors [150]. Some pathogenic bacteria themselves may undergo genetic mutations during the infection process, thereby transforming into non-pathogenic endophytes [151]. Recent studies found that SsHADV-1 could downregulate the expression of key pathogenicity factor genes in *S. sclerotiorum* during infection. Some of the downregulated expression targeted almost all of the cellulose, most hemicellulose-related *PCWDE* genes, approximately half the pectin- and lignin-related *PCWDE* genes, and effectors or effector-like small secretory proteins such as Ss-Cmu1, Ss-ITL, SsSSVP1, SsCP1, Ss-Rhs1, and ssv263 [152]. Biological entities conform to complex patterns. Variation in the lifestyle of systemic endophytes is still in an elusive state and would require comprehensive efforts targeting specific endophytic interaction not only with other host plants but also with other phyla of organisms such as endophyte-infecting viruses.

### 4.6. Plant Host Genetic Loci Regulating Endophytes

Most recently, exciting findings revealed that host genetic loci regulate the diversity and abundance of endophytes [82]. Microbial communities under the influence of host genes serve as core microorganisms to form different ecologies and networks that play a role in the symbiosis of other microorganisms with host plants [124,132,153]. These core microorganisms are present in nearly all communities of the specific plants to which they are associated [154].

The relationship between Arabidopsis plant genes and endophytic microorganisms was investigated through the induction of artificial mutations, revealing that plants exert control over the endophytic microbiota to ensure optimal plant health [155]. Related research has been conducted using the method of artificially creating genetic variation materials in crop plants such as barley [156], soybeans [155,157], and rice [11]. The symbiotic relationship between *Lotus japonicus* and nitrogen-fixing rhizobia is modulated by nodulation pathway genes, with nodulation factor receptor 5 (*NFR5*), lotus histidine kinase 1 (*LHK1*), and nodulation inception (*NIN*) playing key roles in shaping the composition of root and rhizosphere bacterial communities [157]. In rice, the gene encoding cell wall-associated kinase XA4, which is involved in cellulose accumulation, also exerted an influence on the abundance of core microorganisms in rice [139]. Notably, Zhang et al. discovered that the gene *NRT1.1B* in rice, which encodes a nitrate transporter and sensor, is associated with the recruitment of numerous nitrogen cycle-related bacteria that are more abundant in indica rice. These nitrogen cycle-related bacteria facilitate the efficient absorption of soil nitrogen by rice roots, thereby enhancing plant growth and yield [11]. The results demonstrate the intersection between the plant-regulated microbiome and the host plant traits, highlighting the collaborative interaction between plants and their associated microorganisms under the regulatory control of these pivotal plant genes. In future breeding strategies informed by microbial engineering, these pivotal genes offer potential avenues for enhancing plant phenotypes through the modulation of the plant endophytes, thereby promoting agricultural sustainability.

However, the effects of host plant traits on endophyte community composition are generally complex and may be co-regulated by a large number of genes, and a new large-scale and unbiased method is needed to identify host plant effects on endophyte community composition. Plant host-related microbiomes can be used as traits for GWAS to identify host genetic loci related to regulating microbial composition [158,159,160,161,162,163]. To date, a series of GWAS studies have identified associations between the community composition and population abundance of endophytes and plant genotypes across various plant species, such as Arabidopsis, *Panicum virgatum* L., and maize, and have pinpointed several significant quantitative trait loci (QTL) involved in regulating endophytes composition. These QTLs are either involved in cell wall integrity, plant immunity, or the development of leaves and floral organs, root and root hair development, short-chain carbon metabolism, and nitrotoluene metabolism pathways [11,157,158,159]. Significant single nucleotide polymorphisms (SNPs) discovered through GWAS are located within the candidate gene *GLUCANSYN-THASE-LIKE 11* (*GSL11*), which controls defensive mechanisms and maintains the integrity of the cell wall, based on fungal and bacterial characteristics seen on Arabidopsis leaves [148]. In a separate study, the leaf microbiota of 200 Arabidopsis genotypes were investigated in eight field experiments, identifying two loci with a significant association, both for the same microbial hub [159]. The single-nucleotide polymorphisms (SNPs) at these two significant loci are located between *YUC-1* (AT4G32540), which is involved in auxin biosynthesis, and *LEUNIG* (AT4G32551), which is associated with leaf and flower organ development. Notably, there is a significant overlap between the root-colonized microbial community regulated by sorghum genes and the genetically controlled microbial community in maize [132]. There is the existence of an overlap between a QTL controlling plant traits and the diversity of the endophyte community in the host plants. After analyzing the ribosomal DNA diversity of leaf epiphytic bacteria in a maize RIL mapping population, it was found that the QTL regulating leaf Simpson bacterial diversity is related to the regulation of corn leaf spot disease [164]. The preference for host plants is observed in root endophytic microbial communities, and this preference is primarily influenced by host plant immunomodulators, MAMP receptor kinases, and ethylene response factors [165]. Currently, it has been found that host plant traits affecting the construction of the endophyte community are generally controlled by polygenic genes with less of an impact, and this trait is sensitive to the external environment. Additionally, the root microbiota is influenced by plant epigenetic factors, which regulate genome stability and gene sequence transcription, thereby governing numerous vital biological processes [166,167]. For instance, RNA-directed DNA methylation (RdDM) can affect the abundance of the root microbiota [167]. A loss of dicer-like proteins (DCL3, DCL2, and DCL4) in the canonical RdDM pathway will significantly change the abundance of root-associated endophytes, whereas other dicer-like proteins affect the root-associated endophytes in an RdDM-independent manner. These studies provide a reference for further understanding the relationship between plant genotypes and endophytes to provide effective breeding strategies.

## 5. Host–Endophyte–Pathogen Interaction

### 5.1. Host–Endophyte–Pathogen Triangle Concept

**Figure 2:**
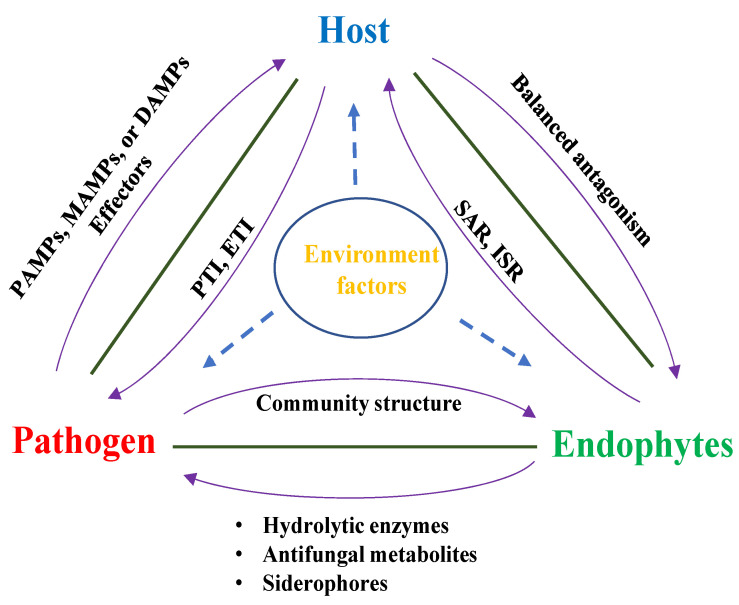
Illustration of the tripartite host-endophyte-pathogen relationship including vital elements of the onset and persistence of specific diseases or their suppression. Endophytes induce systemic acquired resistance (SAR)/ induced systemic resistance (ISR) by host plants, which in turn manifests a balanced antagonism to ensure the endophyte population remains at the optimum. Pathogens potentially evade the host immune system; however, DAMPS, MAMPS, PAMPS, and pathogen effectors trigger PTI or ETI immune responses. The role of the environment acts tridirectionally, impacting endophytes, pathogens, and the host plant during disease occurrence. PTI, PAMP-triggered immunity; ETI, effector-triggered immunity; MAMPs, microbe-associated molecular patterns; PAMPs, pathogen-associated molecular patterns; PTI, PAMP-triggered immunity; DAMPs, damage-associated molecular patterns; ISR, induced systemic resistance; SAR, systemic acquired resistance.

A “disease triangle” theory was put forth by Russell B. Stevens in the 1960s [168]. In the traditional “disease triangle” theory, the occurrence of plant diseases only involves the influence of three factors: plants, pathogens, and the environment. However, a new series of studies suggested that plant endophytes play a fourth role in the disease triangle (Figure 2). In short, a disease triangle consisting of pathogens, endophytes, and host plants forms a distinct relationship that differs from the traditional concept of the disease triangle, comprising vital elements of the onset and persistence of specific disease suppression [19,169,170]. The presence of endophytes can enhance plants’ resistance to pathogen invasion, either through direct or indirect mechanisms [65,171]. It is worth noting that symbiotic microorganisms (including fungi and bacteria) coexist with pathogenic microbes, and these endophytes can help pathogenic microbes promote the occurrence of host plant diseases [172,173]. Microbial resources are utilized by both the “defending” plants and the “attacking” pathogenic microbes to achieve their own goals in their continuous evolution (Figure 2). Understanding host-pathogen-endophyte interaction is key to enriching host plant microbiota aimed at the biocontrol of plant pathogens [174]. The analysis of genetic regulation in the host-pathogen-endophyte disease triangle is therefore of utmost importance.

### 5.2. The Endophyte Functions as an Extension of the Plant’s Immune System

Plants may actively recruit microbial communities that can antagonize pathogenic microbes early during their growth, and the shaping of these microbial communities may be affected by host genes (see Section 3). The activation of host immune responses by endophytes can enhance the host’s resistance to pathogens [170,175]. When external pathogens invade plants, there are changes in the microbial communities in different parts of the plant. This may be because pathogens and endophytes compete for survival and the acquisition of resources, which thereafter affects the abundance of endophytic microorganisms and the stability of the community structure [12,176]. At the same time, the interactions between microorganisms existing in plants either through antagonism or synergism can also affect the occurrence of plant diseases [177,178,179] (Figure 2). There is a significant correlation between the composition of the non-pathogenic endophyte community and the severity of host disease symptoms [174]. The endogenous microbial community in symbiosis with the host plant may have disease-modifying ecological functions, especially the foliar fungal community; for example, the endophytic bacterium *Sphingomonas* discovered in the study of endophytes on Arabidopsis leaves can serve as the plant’s outermost line of defense against attacks by Arabidopsis pathogens [179]. In addition to the early recruitment of beneficial endophytes, when the fungal pathogen *Rhizoctonia solani* infects roots, plants can enhance the abundance of *Chitinaceae* and *Flavobacteriaceae* in root endospheres through the upregulation of the host gene [170]. The rich microbial community formed by these two bacteria can effectively suppress the incidence of fungal root diseases. On the other hand, there is a lot of evidence that endophytes can activate plant ISR [65,66]. The regulation of defense hormones and amino acids (aspartic acid, glutamic acid, serine, and proline) by seed-borne endophytic *B. amyloliquefaciens* RWL-1 facilitates and enhances disease resistance [56]. It is evident that, in addition to secreting ISR-inducing metabolites or inducing defense hormones, endophytes can also bolster systemic resistance in plants by stimulating the production of defense enzymes, such as phenyl ammonia lyase (PAL) [179]. In line with this, the most recent findings have been reported in a recent Arabidopsis study, which suggested the existence of microbiota-dependent autoimmunity and microbiota-independent autoimmunity in plants [169].

## 6. Prospects of Endophytes in Sustainable Agricultural Development

In recent years, extensive researches have been conducted on the intricate interplay between plants and microorganisms. Despite the persisting unresolved inquiries, our comprehension of this interactive system has progressively deepened. Endophyte colonization into host plant tissue is not only different from the process of pathogenic bacteria invading plants but also differs between different endophytes. It is a two-way selection process, which is affected by both the “host” and “guest”. Under the influence of the host, pathogens and endophytes can also undergo reciprocal transformation, which is bidirectional and dynamic, and is also affected by multiple factors. This provides new ideas for the subsequent use of microbial resources to improve crop traits.

A large number of studies have revealed how host factors play an active role in the complex interactions between plants and endophytes at different stages. Based on current research results, it can be speculated that during the interaction between host plants and endophytes, plants have continuously evolved diverse metabolic pathways and even specialized metabolisms to enhance communication with their symbiotic “guests” within their bodies. This process can help plants to shape the internal microbial community according to their specific requirements. However, it remains unclear which specific plant-produced metabolites are involved in regulating microorganisms. Furthermore, the presence of a significant number of uncharacterized biosynthetic genes in plant genomes indicates that there is still much progress to be made in fully comprehending the intricate interplay between plants and their endophyte. To understand the network of interactions between host plants and their endophytic microbiota, it is prudent to determine whether or not a plant’s endophytic microbiota is highly heritable and if there is any existence of community-level selection between the host and endophyte communities or different endophyte-endophyte community members, and more work is needed to uncover the genetic and physiological mechanisms underlying the effects of host plants on symbiotic microbiota. This requires researchers to consider the relationship between endophytes and host plants from a genetic perspective and to investigate plant genes related to plant–microbe interactions.

Currently, the primary approaches employed for mining plant genes associated with plant–microbial interactions encompass GWAS and the generation of genetically mutated plants through artificial means. GWAS can elucidate the impact of host genetic variations on the abundance and diversity of plant endophytic microbial communities, as well as their associations with plant phenotypes and microbial assembly/functioning. Moreover, GWAS enables the prediction of the host’s endophytic microbiome structure based on genotypes. Artificially created genetic variations can also be used to identify the relationship between genetic loci associated with host endophyte community structure. Moreover, it is possible to combine CRISPR/Cas9 technology with comparative genomics technology to target different genes in host plants that control endophyte colonization and community structure through multiple simultaneous gene edits. Compared with GWAS, this method of using individual mutation materials to identify microbial-related sites is less efficient. Additionally, molecular modeling tools have been used to understand microbial enzymes and similar proteins, but proteins involved in plant-microbe interactions have been studied less [180]. Advanced genome-editing tools have a low cost, are reliable, and high-throughput gene sequencing has enabled genomics and metagenomics approaches to study various aspects of organisms and their environments [181]. In short, both methods have their advantages and disadvantages and are complementary to each other. Breeders can comprehensively choose appropriate methods based on their experimental purposes to explore the host plant genome regulating the microbiome.

Endophyte communities differ in different plant tissues [182], so targeting endophyte regulation within plants and research on the composition of microbial communities may require specific tissue targeting. Researchers are currently working extensively in the field to determine host factors that affect the construction of microbial communities. Shaping the composition and function of endophytes or rhizosphere microbial communities by manipulating host genetic variation is one of the major futuristic frontiers targeting the maximum exploitation of endophytes for crop improvement. It is still challenging to cultivate varieties that can influence the composition of microbial communities and thereby improve traits through plant breeding since the regulatory pathways involved are not yet fully elucidated at the molecular level. The identification of core microorganisms regulated by host plants can provide a basis for the artificial construction of SynComs to explore the functions of microbiota when interacting with the host and “arm” the host plants comprehensively to enhance plant production and disease resistance, therefore, providing new feasible solutions for the development of green and sustainable agriculture [183,184,185].

## Data Availability

Not applicable.
